# Biosurfactant Production by *Bacillus salmalaya* for Lubricating Oil Solubilization and Biodegradation

**DOI:** 10.3390/ijerph120809848

**Published:** 2015-08-19

**Authors:** Arezoo Dadrasnia, Salmah Ismail

**Affiliations:** Department of Biohealth Science, Institute of Biological Sciences, Faculty of Science, University of Malaya, Kuala Lumpur 50603, Malaysia; E-Mail: are.dadrasnia@gmail.com

**Keywords:** biosurfactant, bioremediation, lubricating oil, solubilization activity

## Abstract

This study investigated the capability of a biosurfactant produced by a novel strain of *Bacillus salmalaya* to enhance the biodegradation rates and bioavailability of organic contaminants. The biosurfactant produced by cultured strain 139SI showed high physicochemical properties and surface activity in the selected medium. The biosurfactant exhibited a high emulsification index and a positive result in the drop collapse test, with the results demonstrating the wetting activity of the biosurfactant and its potential to produce surface-active molecules. Strain 139SI can significantly reduce the surface tension (ST) from 70.5 to 27 mN/m, with a critical micelle concentration of 0.4%. Moreover, lubricating oil at 2% (v/v) was degraded on Day 20 (71.5). Furthermore, the biosurfactant demonstrated high stability at different ranges of salinity, pH, and temperature. Overall, the results indicated the potential use of *B. salmalaya* 139SI in environmental remediation processes.

## 1. Introduction

Petroleum consumption is rapidly increasing in industry, and as a result, large amounts of hydrocarbons are discharged annually into the environment, either accidentally or deliberately. In addition, many small spills occur during oil recovery, transport, and refining [1]. Recently, the development and implementation of innovative and environmentally friendly technologies to degrade and/or remove these organic compounds have gained increasing attention. Many microbial species are reported to be effective in the breakdown of organic compounds, which are used as energy sources by these microorganisms. Moreover, the use of biocompounds has emerged as a promising method to enhance and improve the effectiveness of bioremediation processes [2,3,4,5]. In this model, biosurfactant molecules act as mediators that increase the mass transfer rate by making hydrophobic pollutants more bioavailable to microorganisms and may also induce changes in the properties of cellular membranes, resulting in increased microbial adherence [4]. This mechanism is of importance when two immiscible phases (oil and water) are present and direct substrate uptake is plausible.

Biosurfactants or surface-active agents are amphipathic molecules comprising an important class of chemical compounds [6]. Such biosurfactants are biologically extracted as components of the bacterial or yeast cell membrane; they have low toxicity and are biodegradable because they can reduce interfacial tension, surface tension (ST), and the critical micelle concentration (CMC). Furthermore, these biocompounds can survive in a wide range of pH and temperature conditions and to some extent can affect interfaces [7]. In addition, because biosurfactants consist of naturally occurring molecules, such as lipopeptides, glycolipids, fatty acids, and lipoproteins, they are more suitable for environmental and petrochemical applications than synthetic and traditional chemical surfactants [8,9]. Bacterial strains belonging to genera *Bacillus* and *Pseudomonas* typically produce lipopeptide biosurfactants; Table 1 shows the main types of biosurfactants produced by some microorganisms [10]. Two mechanisms can be employed to enhance hydrocarbon degradation with biosurfactants: (1) improving the transfer of contaminants into the aqueous phase by interaction with soluble contaminants; and (2) increasing the availability and solubility of hydrocarbons by reducing ST. These mechanisms allow contact of the bacterial cell surface with hydrophobic substrates [2].

**Table 1:** Microbial biosurfactants.

Microorganism	Biosurfactant
*Pseudomonas sp.*	Rhamnolipids
*Rhodococcus sp.*	Trehalose lipids
*Candida sp.*	Mannosyl erythritol lipid
*Candida bogoriensis*	Sophorolipid
*Corynebacterium lepus*	Corynemycolic acids
*Candida petrophilum*	Peptidolipid
*Bacillus subtilis*	Cyclic lipopeptide
*Bacillus licheniformis*	Cyclic lipopeptide
*Candida tropicalis*	Mannan-fatty acid complex

Many microbial ecologists have reported the potential of various *Bacillus* species to produce good biosurfactants under aerobic and anaerobic conditions that can be used in the food industry [8,11]. From an economic perspective, the application of low-cost substrates is an important factor for successfully developing biosurfactant production.

The present study aimed to evaluate the stability of a biosurfactant produced by *B. salmalaya* 139SI under different environmental conditions and to characterize its properties. This study also investigated the capacity of the biosurfactant to solubilize and facilitate the degradation of oil and to reduce ST at different environmental conditions. Most importantly, the ability of the biosurfactant to biodegrade lubricating oil from contaminated water was evaluated.

## 2. Materials and Methods

### 2.1. Microorganism Isolation

Bacterial cells were harvested, and genomic DNA was extracted using NucleoSpin Tissue in accordance with the manufacturer’s instructions. Selected 16S rRNA universal primers, namely, 27Forward (5′-AGAGTTTGATCMTGGCTCAG-3′) and 1492Reverse (5′- GGTTACCTTGTTACGACTT -3′), were used to amplify the 16S rRNA region [12].

### 2.2. Culture Medium and Biosurfactant Preparation

*Bacillus salmalaya* 139SI was cultured in 1 L of brain–heart infusion (BHI) medium containing 5 g/L KCl, 3 g/L dextrose, 2.5 g/L Na_2_HPO_4_, 14.5 g/L gelatin, 6 g/L BHI, and 6 g/L peptic digest of animal tissue in a shaking incubator at 150 rpm for 72 h at 35 °C. The biosurfactant was produced using the method described by Rufino *et al.*, [7,13]. The 72 h culture was centrifuged (8000 × g for 15 min) and filtered through a Whatman no.1 filter. The cell-free broth was concentrated by freeze-drying (stored at −20 °C) and extracted twice with chloroform in a separatory funnel at 28 °C. For measurement of carbohydrates (reducing sugars), a microtiter plate adaptation of the dinitrosalicylic acid (DNS) colorimetric method was used [14]. The protein concentration of the isolated biosurfactant was estimated using the DC Protein Assay Reagents Package™ (BioRad). Different parameters, such as pH, biosurfactant production, growth kinetics, and ST, were monitored at regular intervals.

### 2.3. Determination of Biomass

Approximately 10 mL of the sample was transferred to a pre-weighted tube and centrifuged at 8000 rpm for 30 min. The cell pellets were washed twice and dried for 48 h at 90 °C. All assays were performed in triplicate.

### 2.4. Drop Collapse and Oil Displacement Tests

Drop collapse and oil displacement tests were conducted using the method described by Ayed *et al.* [2]. Briefly, 2 µL of lubricating oil was added to 96-well plates. After 1 h equilibrium at 35 °C, 5 µL of the supernatant obtained from the culture was added to the oil surface. Distilled water was used as control. After 1 min, the shape of the drop was observed. Bead-like and collapsed drops indicated negative and positive results, respectively. To measure the clear zone diameter, an oil displacement test was performed by dropping 20 µL of oil onto 50 mL of distilled water in a Petri dish followed by the addition of 10 µL of the supernatant. The clear zone diameter was determined and compared with that of the control.

### 2.5. Determination of Emulsification Activity

The emulsification index (EI_24_) was measured using a previously described method [15], with slight modifications, at 25 °C by vortexing 4 mL of the biosurfactant and lubricating oil for 5 min. After 24 h, EI was calculated as follows:

EI_24_ (%) = *α* / *β* ×100 (1)

where *α* and *β* represent the height of the emulsified layer and the total height, respectively.

### 2.6. Determination of Bacterial Growth and Surface Tension Reduction

After 7 days of incubation, bacterial growth was determined via spectroscopy at 600 nm to assess nutrient and physical parameters. Cultures were centrifuged at 8000 rpm for 30 min at 4 °C, and the cell-free supernatants were used to determine ST with a tensiometer (mN/m). The ST percentage was calculated as follows [16]:

ST reduction (%) = Sc − Ss/Sc × 100(2)

where Sc is the ST of the control and Ss is the ST of the sample.

### 2.7. Critical Micelle Concentration of the Biosurfactant

Dilutions of the biosurfactant in distillate water were prepared to determine CMC up to a constant ST value. CMC was determined as mg/L by plotting the ST concentration against the ST value.

### 2.8. Fourier Transform Infrared Spectroscopy

To understand the overall chemical nature of the extracted biosurfactant, Fourier transform infrared spectroscopy (FTIR) was employed, which helps to explore the functional groups and chemical bonds present in a crude extract. The analysis was performed using spectrum 4000, US. Samples were prepared via homogeneous dispersal of 1 mg of the biosurfactant sample in pellets of potassium bromide powder at a ratio of 1:100 and pressed under 10 tons of pressure. IR spectra were collected over the 600 and 4000 cm^−1^ range. 

### 2.9. Stability Analyses

*Du Nouy* ring methodology was used to measure ST (KSV Sigma 702, Finland); the ST of deionized water was also measured (70.5 mN/m) to calibrate the tensiometer. Up to 25 mL of each surfactant solution was placed in a clean beaker, and ST was measured using a platinum wire ring. To increase the accuracy of the experiment, the ST measurements were performed at room temperature (22 °C).

#### 2.9.1. pH and Temperature

Approximately 10% (v/v) of inoculum was incubated in a 100 mL flask containing 50 mL of BHI and incubated at 35 °C in an orbital shaker at 150 rpm for 3 days. Different pH values (2 to 10) were used to determine the optimal pH. Afterward, the inoculum was adjusted at an optimized pH following incubation at 200 rpm for 7 days at different temperatures (20 °C to 50 °C).

#### 2.9.2. Carbon, Nitrogen, and Phosphorus Sources

The effects of various carbon sources (olive, sunflower, transformer, glycerol, and vegetable oils) on bacterial growth and biosurfactant production were investigated. A final concentration of 1% (v/v) of each source was sterilized and filtered (0.2 µm pore size) and then added separately to evaluate the effects on biosurfactant production. The cultures were placed in a shaking incubator at 200 rpm for 7 days at 35 °C. The effect of salinity on ST was tested by fixing the optimized pH value and adjusting different concentrations of NaH_2_PO_4_ (0, 7, and 15 g/L), (NH_4_)_2_SO_4_ (0, 1, and 2 g/L), and NaCl (20, 40, and 60 g/L).

### 2.10. Lubricating Oil Solubilization Test

A solubility assay for lubricating oil was performed in the presence of different biosurfactant concentrations (0.1 g/L to 2.4 g/L). Up to 300 μL of lubricating oil was added (to a final volume of 30 mL) to the biosurfactant in test tubes containing HCl buffer. The tubes were capped and incubated in an orbital shaker (150 rpm at 35 °C). After 24 h, the residual oil at the surface of the tubes was separated and transferred to clean tubes containing the same amount of n-hexane. The tubes were vortexed for 3 min and centrifuged for 20 min at 80,000 rpm. The oil concentration was measured at 300 nm. Control samples, which contained no biosurfactant, were subjected to the same method. All assays were performed in triplicate.

Percentage of solubilization = C_lubricating oil_ – C_control_/C_inital_ × 100 (3)

where C_lubricating oil_ is the oil concentration after extraction from the hexane solution; C_control_ is the oil concentration in the control: and C_initial_ is the oil concentration prior to the solubilization assay.

#### Lubricating Oil Degradation Assay

The biosurfactant produced by *B. salmalaya* 139SI was examined to determine the biodegradation rate. Strain 139SI was grown in BHI broth (150 rpm at 37 °C) to achieve OD_600_ of 1, and 10% (w/v) was used as seed culture for biosurfactant production. The bacterial cells were harvested by centrifugation and resuspended in sterilized mineral salt medium (MSM) containing 1.8 g K_2_HPO_4_, 0.1 g NaCl, 1.2 g KH_2_PO_4_, 0.01 g FeSO_4_.7H_2_O, 4 g NH_4_Cl, and 0.2 g MgSO_4_.7H_2_O. To assess the biodegradation of lubricating oil (2% v/v), inoculum (1 g/L of strain 139SI) was added to 50 mL of sterilized MSM.

Uninoculated medium was used for the control treatment. The flasks were incubated in a shaking incubator maintained at 35 °C for 24 days at 150 rpm. The residual oil was extracted twice using 50 mL of n-hexane as the solvent and then dried with anhydrous sodium sulfate. The solvent was evaporated using a rotary evaporator, and the weight of the residual oil was measured and recorded. The percentage of oil degradation was calculated as follows:

YPH degradation (%) = TC – TT/TC × 100(4)

where TPH is the total petroleum hydrocarbon, TC is the TPH in the control sample, and TT is the TPH in the treatment. Additionally, the extracted hydrocarbon was analyzed by gas chromatography with a helium carrier gas flow of 1.27 mL/min. The column oven was initially held at 50 °C for 2 min, increased to 300 °C at a rate of 6 °C min^−1^, and held for 16 min.

### 2.11. Statistical Analyses

The means of the treatments were evaluated by ANOVA using a general linear model (SPSS 18). Duncan’s multiple range tests were performed to determine the level of significance (*p* < 0.05).

## 3. Results and Discussion

### 3.1. Biosurfactant Activity Assay Tests

**Figure 1:**
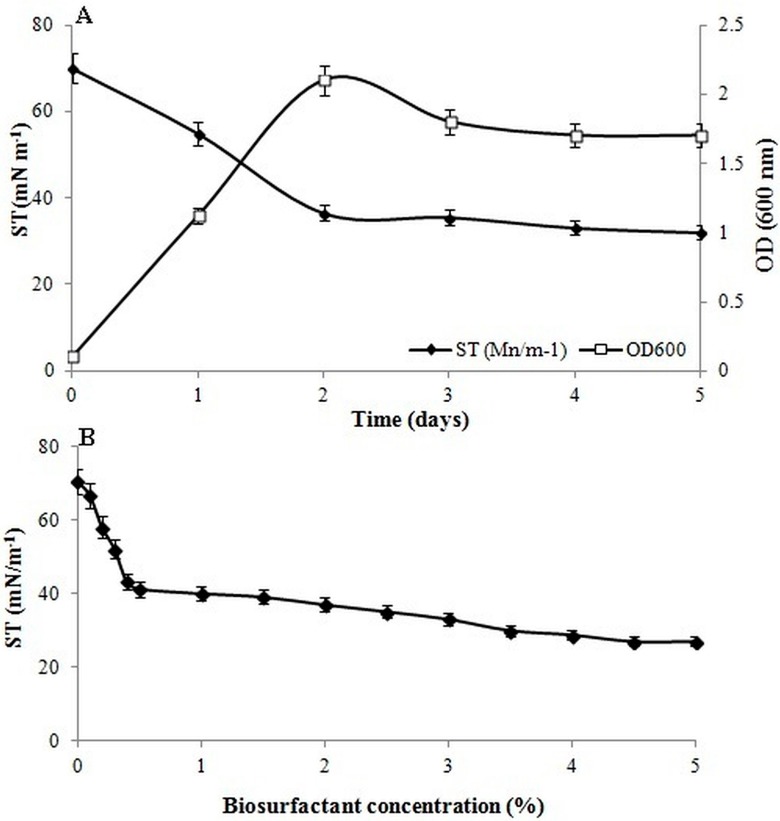
(**A**) Surface tension reduction and time course of cell growth in brain–heart infusion (BHI) medium. (**B**) Surface tension *versus* concentration of biosurfactant produced by *B. salmalaya* 139SI. Vertical bars indicate SE (*n* = 3).

Drop collapse and oil spreading techniques were used because of their low cost, simplicity, and quick implementation. The positive result obtained in the drop collapse test revealed the wetting activity of the biosurfactant and its potential to produce surface-active molecules. The oil displacement test is an index to predict the surfactant production caused by the decrease in the oil-water interfacial tension [2], and the area of oil displacement (75 cm^2^) indicated high activity of the biosurfactant produced by *B. salmalaya* 139SI. The emulsification index (% E_24_) of 139SI decreased at low pH, and the highest emulsification (65 ± 1.1%) was observed between pH 5 and 8. Cunha *et al.*, [17] used gasoline as a carbon source to produce a biosurfactant during fermentation at pH 6.0. Each microorganism is adapted to a specific pH, depending on the relevant type of biosurfactant, and such specificity facilitates the bioavailability of organic compounds to bacteria, accelerating hydrocarbon degradation. Additionally, the emulsion activity of strain 139SI was stable for two weeks at room temperature. The profiles of biomass at 600 nm and ST during five days of incubation time are shown in Figure 1A; growth was carried out at 32 °C and 150 rpm. The ST of the biosurfactant rapidly decreased after cultivation and became constant after 48 h (36.5 mN/m). Maximum growth was observed after two days, followed by a steady decrease until the end of the incubation (120 h). Furthermore, several authors have reported that the fluid ST did not vary once micelle formation began. Ruggeri *et al.*, [18] indicated that quantitative analyses, including ST determination, are reliable methods for determining the solubility of biosurfactants in media. If ST is reduced to <40 mN/m and/or at least 50% emulsification is observed after 24 h, the strain can be considered to be a biosurfactant producer. Nitschke and Pastore [19] reported that *B. subtilis* is one of the most effective *Bacillus* species in this regard because it can reduce ST from 72 to 27 mN/m. In the present study, *B. salmalaya* 139SI showed positive results in all the quantitative tests. The carbohydrate and protein contents of strain 139SI were 0.72 g/L and 1.9 g/L, respectively.

### 3.2. Critical Micelle Concentration

To establish the CMC of the purified biosurfactant from *B. salmalaya* 139SI, the relationship between biosurfactant concentration and surface tension was determined (Figure 1B). The surface activity of a biosurfactant depends on its ability to decrease the formation of a stable emulsion, as well as its ST and CMC values [20]. The minimum biosurfactant concentration or CMC should be determined in order to obtain maximum ST reduction. With increasing biosurfactant concentration, ST was significantly reduced from 70.5 mN/m to 27 mN/m and then remained constant to a CMC value of 5%. This CMC value of the biosurfactant produced by *B. salmalaya* 139SI was much lower than that of a chemical surfactant, which indicated that the CMC was reached. The length of the fatty acid chain also affects CMC, with a low CMC being caused by long-chain fatty acids [21]. The reported CMC values for different types of biosurfactants produced by *Bacillus* species are within this range [22,23,24]. *Bacillus* show smaller CMC and ST values than other genera, such as *Candida* and *Pseudomonas*. Ayed *et al.*, [2] reported the minimum ST and CMC values for *B. amyloliquefaciens* An6 (29 mN/m), and the biosurfactants produced by *B. subtilis* B20 and *B. subtilis* BS-37 were able to reduce the ST and interfacial tension to 25 and 28 mN/m, respectively [2,22].

### 3.3. Fourier Transform Infrared Spectroscopy

**Figure 2:**
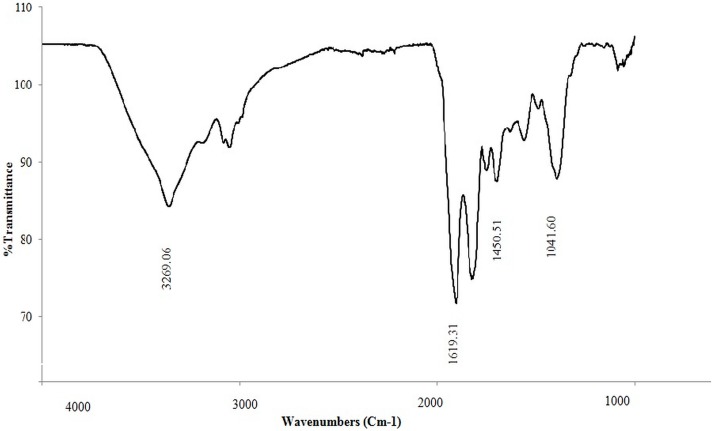
Fourier transform infrared spectra (FTIR) of the biosurfactant produced by *Bacillus salmalaya.*

As a result of C–H stretching vibrations, a broad absorbance peak with variable wave numbers was observed (Figure 2). This is typical of carbon compounds containing amino groups. The sharp absorbance peaks at 1041 and 1450 cm^−1^ are indicative of aliphatic chains (–CH_3_ and –CH_2_–), and these peaks reflect the presence of alkyl chains in a compound. The peaks at approximately 3269 cm^−1^ indicate the C–H bonds of –CH_2_ groups. The strong band observed at 1619.31 cm^−1^ is attributed to a carbonyl group; the presence of C=O bonds causing C=O stretching vibrations, which leads to absorbance peaks in these regions. The FTIR spectrum suggests the production of a lipopeptide biosurfactant.

### 3.4. Stability Studies

**Figure 3:**
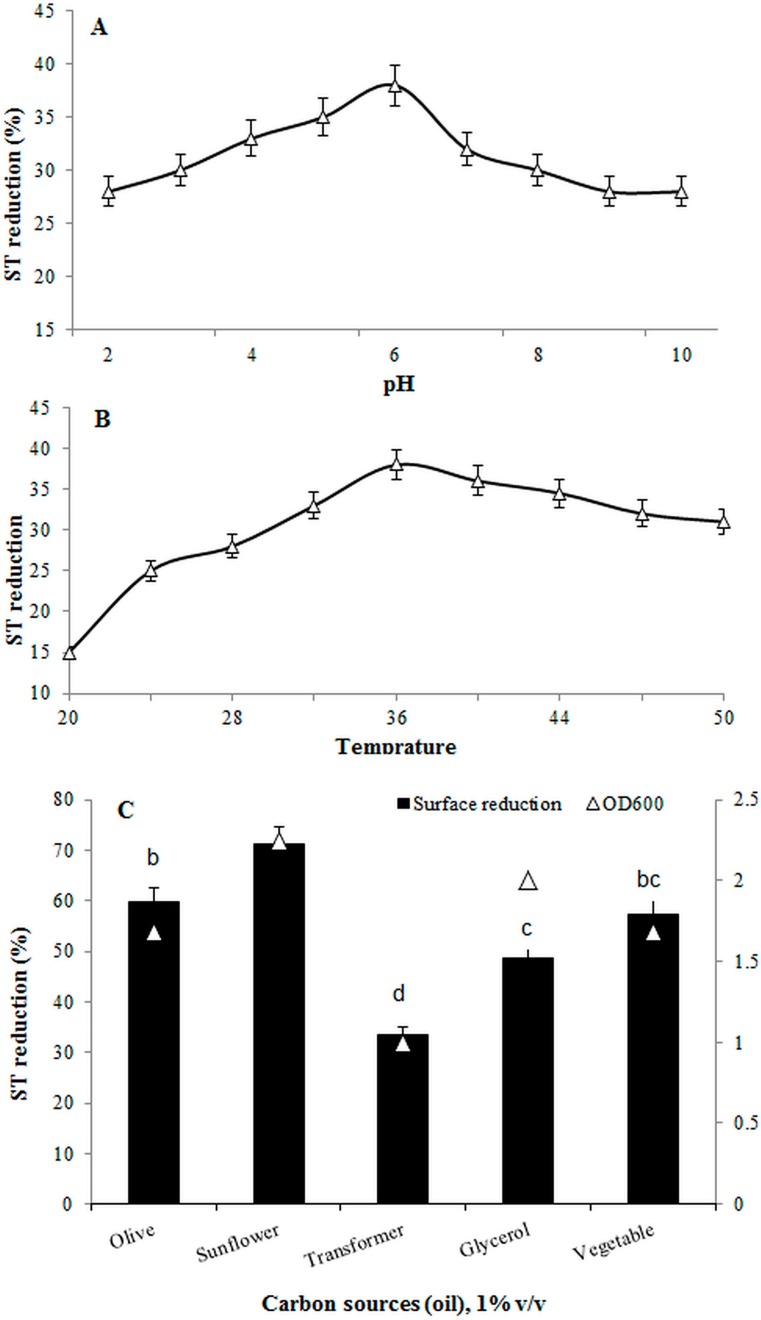
Influence of different ranges of (**A**) pH, (**B**) temperature and, (**C**) carbon source on surface tension (ST) reduction.

As many factors influence the effectiveness of biosurfactant activities, these parameters must be examined to confirm the application of a biosurfactant during remediation. In the present study, a wide range of pH and temperature was tested to evaluate the stability of *B. salmalaya* 139SI to grow and produce biosurfactants. As shown in Figure 3A, a pH of 6.5 was the optimal value for biosurfactant production with the highest rate of ST reduction (38.5 ± 2.1%) when compared with the treatment without inoculation. The results also showed a low level of biosurfactant production at acidic pH (<5) and alkaline pH (>9). Various species produce biosurfactants at different pH values. *Bacillus salmalaya* 139SI produced the maximum amount of biosurfactant at pH 6.5 when incubated at 36 °C, which was significantly different from the production at other temperatures. The lowest ST was produced at 20 °C (Figure 3B); ST sharply increased when the temperature increased up to 36 °C and then gradually decreased and remained constant at 46 °C. Thus, temperature affects biosurfactant production, a finding that is consistent with the results of other studies [3,25,26,27]. Nonetheless, different *Bacillus* strains present various optimal temperatures and pH values. 

**Figure 4:**
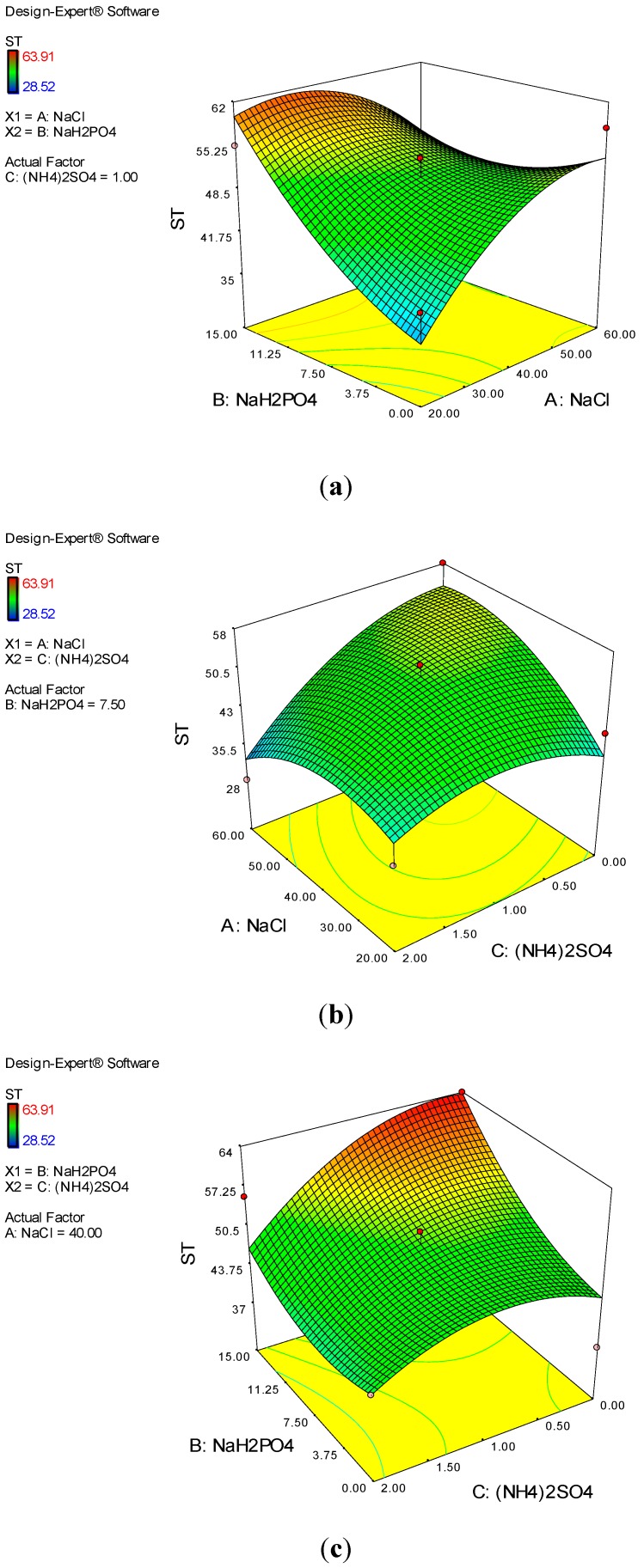
Influence of NaCl (**a**), NaH_2_PO_4_ (**b**) and (NH4)_2_SO_4_ (**c**) on ST reduction (%).

The effect of different carbon sources on ST was evaluated using 1% (v/v) olive, sunflower, transformer, glycerol, and vegetable oils. As shown in Figure 3C, a significant reduction in ST was observed with particular carbon sources. The lowest and highest ST reduction rates were found for the transformer and sunflower oils, with values of 33.5 and 71.1%, respectively (Figure 3C). Moreover, ST decreased with olive, vegetable, and glycerol oils, to 59.6, 57.3, and 48.6%, respectively. This result is in contrast with that of Zhang *et al.* [28] who reported the ability of *P. aeruginosa* to produce a biosurfactant in 30 g/L glycerol compared with paraffin and vegetable oil. In the present study, the reduction of ST in glycerol was less than that in the other carbon sources, which may be ascribed to the different bacterial genera used, and the various optimal pH and temperature values for biosurfactant production. The effects of salt and N and P concentrations on biosurfactant production were also assessed. Given the relatively high salinity in oil refineries, halophilic bacteria should be used during oil recovery. N and P concentrations and the nutrient formulation are important parameters to ensure rapid bacterial growth and biosurfactant production. Therefore, the possibility of biosurfactant production depends on the complex structure of the biosurfactant itself and on the concentrations of salts, N, and P. The results illustrated that the percentage of ST reduction was enhanced when the concentration of NaH_2_PO_4_ as a source of P increased to 15 g/L (Figure 4A); this finding indicated the positive effect of P on biosurfactant production. The maximum ST reduction (63.91%) was observed when 15 g/L NaH_2_PO_4_ and 40 g/L NaCl were added; in contrast, the percentage of ST reduction decreased to 45.9% with the addition of 60 g/L NaCl and 15 g/L NaH_2_PO_4_. This result is in very close agreement with the findings of Huszcza and Burczyk [29], who reported that the biosurfactant activity produced by *B. coagulans* was enhanced with the addition of salts. In addition, the maximum activity of the biosurfactant produced by *B. subtilis* was achieved with the addition of 15% NaCl [30]. The current results also indicated that *B. salmalaya* can grow under conditions of high salinity. The highest percentage of ST reduction (53.3%) with the simultaneous application of NaCl and (NH_4_)_2_PO_4_ was observed at concentrations of 40 and 1 g/L, respectively (Figure 4B). As shown in Figure 4C, to decrease ST, the optimal concentration of NaH_2_PO_4_ was 15 g/L without the addition of (NH_4_)_2_PO_4_. These results showed the inhibiting effect of low and high concentrations of N, P, and salts on the biological activities of strain 139SI. However, the lowest supernatant ST reduction (28.5%) was observed when the concentrations of NaCl, NaH_2_PO_4_, and (NH_4_)_2_PO_4_ were increased to 60, 7.5, and 2 g/L, respectively. Thus, increasing the concentration of NaCl and (NH_4_)_2_PO_4_ caused a decrease in biosurfactant production.

### 3.5. Solubilization Study

**Figure 5:**
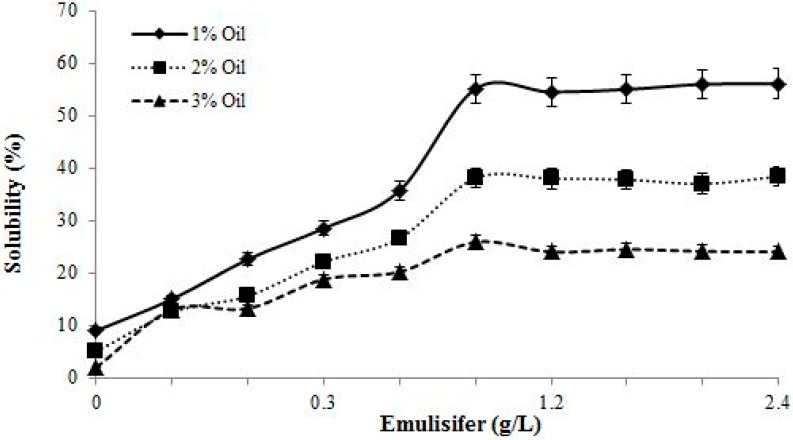
Effect of biosurfactant concentration on oil solubilization.

The enhancement of the solubility of the biosurfactant at different concentrations was evaluated. As shown in Figure 5, at a concentration of 0.8 g/L, the solubility rate gradually increased followed by a sharp increase and then remained constant, regardless of an increase in oil concentration. Furthermore, the solubility rates at different lubricating oil concentrations were significantly different from each other. At the same emulsifier concentration, the solubility rate increased at lower oil concentrations; this result indicated the specific activity of the oil in the solubilization process, which affected the solubility of oil in water. However, the effectiveness of the biosurfactant to enhance water solubility was higher at 1% oil than that at 2 and 3%. Furthermore, the percentage of solubility at a biosurfactant concentration of 2.4 g/L decreased from 56.1 to 24 mN/m when the oil concentration increased from 1 to 3% (Figure 5). This result demonstrated that a high amount of hydrocarbon functions as a limiting factor during solubilization. The effects of different pH values on solubilization activity were also determined. The results showed that the biosurfactant activity decreased in acidic pH, which is in line with the results obtained by Joshi *et al.* [31] who reported biosurfactant precipitation at pH 2.

### 3.6. Biodegradation of Lubricating Oil

**Figure 6:**
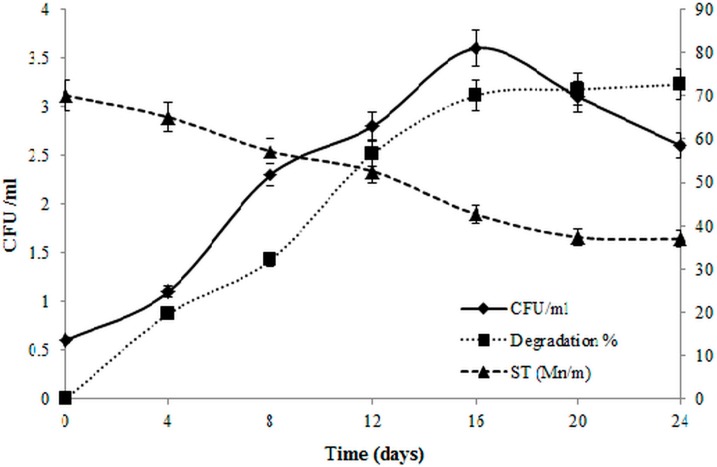
Changes in ST, biodegradation rate and strain growth during 24 days. Vertical bars indicate SE (*n* = 3).

The potential of *B. salmalaya* 139SI to utilize lubricating oil (2% v/v) as a source of energy and carbon was investigated for 24 days. Cultures were incubated at 35 °C, and samples were collected every four days to monitor the rates of oil degradation, microbial population, and ST. As shown in Figure 6, the strain was able to grow with 2% oil. The rate of oil degradation was determined via a gravimetric method, and the results illustrated that 19.6% of the oil was degraded during the first four days, with the maximum rate of degradation of 72.6% on Day 24 (Figure 6). After 20 days of incubation, the rate of oil degradation remained constant. The number of colony-forming units increased up to Day 16 (3.6 × 10^6^), followed by a decrease until the end of the experiment; the value was up to 2.6 × 10^6^ on Day 24. The biosynthesis of the biosurfactant was determined by measuring ST. The amount of ST slowly decreased from 70.1 mN/m on Day 0 to 52.6 mN/m on Day 12, and the maximum reduction was observed on Day 20, reaching 37.4 mN/m. Many studies have reported the ability of microbial surfactants to degrade hydrocarbons. In our previous study, we reported the potential of *B. salmalaya* 139SI for use in the bioremediation of crude oil waste [3,12]. Ayed *et al.*, [2] reported that the biodegradation of diesel oil was enhanced by the addition of *B. amyloliquefaciens* An6 compared with an anionic surfactant and Tween 80. Therefore, decreasing ST with the addition of a biosurfactant at the water-air interface enhances and improves solubility, thereby reducing the partitioning of organic carbons from the oil phase into aqueous solution.

## 4. Conclusions

In this study, the optimal pH of the biosurfactant produced by 139SI was found to be 6.5, and the highest ST reduction (71.1%) was observed in a medium containing 1% sunflower oil incubated at 36 °C. This biosurfactant demonstrated high emulsification activity and efficiency under extreme environmental conditions during remediation. Moreover, this biosurfactant likely enhanced the biodegradation and bioavailability of lubricating oil by reducing ST between the bacterial cell surface and oil. The biosurfactant produced by 139SI remained stable under various conditions, exhibited low toxicity, and enhanced the biodegradation of lubricating oil. Overall, the biosurfactant from *B. salmalaya* 139SI presents interesting features and therefore constitutes an alternative with potential use in several industries.

## References

[1] Bolliger C. Bioremediation of a Heating Oil-Contaminated Aquifer-Quantification Of Processes By Chemical, Biological, and Stable Isotope Analyses. http://e-collection.library.ethz.ch/eserv/eth:23731/eth-23731-02.pdf.

[2] Ayed B.H., Jemil N., Maalej H., Bayoudh A., Hmidet N., Nasri M. (2015). Enhancement of solubilization and biodegradation of diesel oil by biosurfactant from *Bacillus amyloliquefaciens* AN6. Int. Biodeter. Biodegr..

[3] Dadrasnia A., Salmah I. (2014). Bio-enrichment of waste crude oil polluted soil: Amended with *Bacillus 139SI* and organic waste. Int. J. Environ. Sci. Dev..

[4] Ławniczak Ł., Marecik R., Łukasz C. (2013). Contributions of biosurfactants to natural or induced bioremediation. Appl. Microbiol. Biotechnol..

[5] Roy A.S., Baruah R., Borah M., Singh A.K., Deka Boruah H.P., Saikia N., Deka M., Dutta N., Chandra Bora T. (2014). Bioremediation potential of native hydrocarbon degrading bacterial strains in crude oil contaminated soil under microcosm study. Int. Biodeter. Biodegr..

[6] Pereira J.F.B., Gudiña E.J., Costa R., Vitorino R., Teixeira J.A., Coutinho J.A.P., Rodrigues L.R. (2013). Optimization and characterization of biosurfactant production by *Bacillus subtilis* isolates towards microbial enhanced oil recovery applications. Fuel.

[7] Rufino R.D., de Luna J.M., de Campos Takaki G.M., Sarubbo L.A. (2014). Characterization and properties of the biosurfactant produced by *Candida lipolytica* UCP 0988. Electron. J. Biotechnol..

[8] Al-Bahry S.N., Al-Wahaibi Y.M., Elshafie A.E., Al-Bemani A.S., Joshi S.J., Al-Makhmari H.S., Al-Sulaimani H.S. (2013). Biosurfactant production by *Bacillus subtilis* b20 using date molasses and its possible application in enhanced oil recovery. Int. Biodeter. Biodegr..

[9] Al-Sulaimani H., Joshi S., Al-Wahaibi Y., Al-Bahry S., Elshafie A., Al-Bemani A. (2011). Microbial biotechnology for enhancing oil recovery: Current developments and future prospects. Biotechnol. Bioinf. Bioeng..

[10] Healy M.G., Devine C.M., Murphy R. (1996). Microbial production of biosurfactants. Resour. Conserv. Recy..

[11] Neves L., de Oliveira K., Kobayashi M., Penna T., Converti A. (2007). Biosurfactant production by cultivation of *Bacillus atrophaeus* ATCC 9372 in semidefined glucose/casein-based media. Appl. Biochem. Biotechnol..

[12] Ismail S., Dadrasnia A. (2015). Biotechnological potential of *Bacillus salmalaya* 139SI: A novel strain for remediating water polluted with crude oil waste. PLoS ONE.

[13] Rufino R., Sarubbo L., Neto B., Campos-Takaki G. (2008). Experimental design for the production of tensio-active agent by *Candida lipolytica*. J. Int. Microbiol. Biotechnol..

[14] Goncalves C., Rodriguez-Jasso R.M. (2010). Adaptation of dinitrosalicylic acid method to microtiter plates. Anal. Methods.

[15] Luna J.M., Rufino R.D., Sarubbo L.A., Campos-Takaki G.M. (2013). Characterisation, surface properties and biological activity of a biosurfactant produced from industrial waste by *Candida sphaerica* UCP0995 for application in the petroleum industry. Colloid Surface. B.

[16] Hamzah A., Sabturani N., Radiman S. (2013). Screening and optimization of biosurfactant production by the hydrocarbon-degrading bacteria. Sains Malays..

[17] Cunha C.D., do Rosário M., Rosado A.S., Leite S.G.F. (2004). *Serratia sp.* Svgg16: A promising biosurfactant producer isolated from tropical soil during growth with ethanol-blended gasoline. Process Biochem..

[18] Ruggeri C., Franzetti A., Bestetti G., Caredda P., La Colla P., Pintus M., Sergi S., Tamburini E. (2009). Isolation and characterisation of surface active compound-producing bacteria from hydrocarbon-contaminated environments. Int. Biodeter. Biodegr..

[19] Nitschke M., Pastore G.M. (2006). Production and properties of a surfactant obtained from *Bacillus subtilis* grown on cassava wastewater. Bioresource Technol..

[20] Bozo-Hurtado L., Rocha C., Malavé R., Suárez P. (2012). Biosurfactant production by marine bacterial isolates from the venezuelan atlantic front. B. Environ. Contam. Tox..

[21] Li Y., Zou A.H., Ye R.Q., Mu B.Z. (2011). Effects of molecular structure on surfactant micellization activity. Acta Phys. Chim. Sin..

[22] Liu Q., Lin J., Wang W., Huang H., Li S. (2015). Production of surfactin isomers by *Bacillus subtili*s BS-37and its applicability to enhanced oil recovery under laboratory conditions. Biochem. Eng. J..

[23] Varadavenkatesan T., Murty V.R. (2013). Production of a lipopeptide biosurfactant by a novel *Bacillus* sp. And its applicability to enhanced oil recovery. ISRN Microb..

[24] Faria A.F., Teodoro-Martinez D.S., de Oliveira Barbosa G.N., Gontijo Vaz B., Serrano Silva Í., Garcia J.S., Tótola M.R., Eberlin M.N., Grossman M., Alves O.L. (2011). Production and structural characterization of surfactin (c14/leu7) produced by *Bacillus subtilis* isolate lsfm-05 grown on raw glycerol from the biodiesel industry. Process Biochem..

[25] Sousa M., Dantas I.T., Feitosa F.X., Alencar A.E.V., Soares S.A., Melo V.M.M., Gonçalves L.R.B., Sant’ana H.B. (2014). Performance of a biosurfactant produced by *Bacillus subtilis* lami005 on the formation of oil / biosurfactant / water emulsion: Study of the phase behaviour of emulsified systems. Braz. J. Chem. Eng..

[26] Moldes A.B., Paradelo R., Vecino X., Cruz J.M., GudiL A.E., Rodrigues L., Teixeira J.A., Domínguez J.M., Barral M.T. (2013). Partial characterization of biosurfactant from *Lactobacillus pentosus* and comparison with sodium dodecyl sulphate for the bioremediation of hydrocarbon contaminated soil. Biomed Res. Int..

[27] Joshi-Navare K., Prabhune A. (2013). A biosurfactant-sophorolipid acts in synergy with antibiotics to enhance their efficiency. Biomed Res. Int..

[28] Zhang G.-l., Wu Y.-t., Qian X.-p., Meng Q. (2005). Biodegradation of crude oil by *Pseudomonas aeruginos*a in the presence of rhamnolipids. J. Zhejiang Univ. Sci. B.

[29] Huszcza E., Burczyk B. (2003). Biosurfactant production by *Bacillus coagulans*. J. Surfactants Deterg..

[30] Mnif I., Ellouze-Chaabouni S., Ghribi D. (2013). Economic production of *Bacillus subtilis* SPB1 biosurfactant using local agro-industrial wastes and its application in enhancing solubility of diesel. J. Chem. Technol. Biot..

[31] Joshi S., Bharucha C., Jha S., Yadav S., Nerurkar A., Desai A.J. (2008). Biosurfactant production using molasses and whey under thermophilic conditions. Bioresource Technol..

